# Energy stress and adaptation strategy of tumor cells in different microenvironments: from primary tumors to distant metastases: Tumor cell adaptation to energy stress in diverse microenvironments

**DOI:** 10.3724/abbs.2025106

**Published:** 2025-07-04

**Authors:** Mingzhe Xu, Junjie Fei, Zhi-Xiong Xiao, Yong Yi

**Affiliations:** Center of Growth Metabolism and Aging Key Laboratory of Bio-Resource and Eco-Environment Ministry of Education College of Life Sciences Sichuan University Chengdu 610064 China

**Keywords:** tumor microenvironment, energy stress, metabolic reprogramming, tumor cell survival

## Abstract

Since the Warburg effect was first described in the 1920s, tumor energy metabolism has been a central focus of cancer research, emerging as a potential therapeutic target. The tumor microenvironment—including blood vessels, immune cells, stromal components, and other cell types—profoundly influences tumor cell metabolism. Variations in energy supply, oxygen availability, nutrient composition, and the accumulation of metabolic waste across different microenvironments challenge tumor cell survival and progression. In response, tumor cells adapt through flexible regulation and reprogramming of metabolic pathways. Although recent studies have explored metabolic adaptation mechanisms in various tumor microenvironments, the full spectrum from primary tumors to distant metastases remains unexplored. This review summarizes energy stress and adaptation maneuvers in tumor cells across different stages of tumor progression and offers a new perspective for comprehensive research to explore therapeutic strategies targeting tumor metabolism.

## Introduction

When normal cells in human tissues acquire mutations that lead to uncontrolled proliferation, tumorigenesis initiates [1]. At the stage of carcinoma *in situ*, tumor cells are confined to their site of origin without breaching the basement membrane or exhibiting invasion or distant metastasis. However, cancer cells are not restricted to the primary site; they possess a certain degree of invasive potential [2]. When carcinoma *in situ* cells undergo epithelial-mesenchymal transition (EMT), they gradually acquire invasiveness, thereby penetrating the basement membrane and entering surrounding tissues. They can then disseminate to distant organs via the bloodstream or lymphatic system in the form of tumor cell clusters, where they colonize and form secondary tumors [3].

Tumor cells face a constantly changing microenvironment and energetic stresses, from carcinoma *in situ* to metastasis via the vasculature or lymphatic system. The foremost challenge is nutrient shortages. The allocation of nutrients in different regions of the human body is limited. This often fails to meet the substantial energy demands of rapidly proliferating tumor cells, thereby impeding their normal metabolism and growth [4]. Moreover, the abundant normal cells surrounding the tumor compete with tumor cells for nutrients, further exacerbating the scarcity of nutrients [5]. Additionally, the metabolic by-products of normal cells can interfere with the metabolic processes of tumor cells. Abnormal vascular distribution leads to a decrease in the transport efficiency of nutrients, which increases the difficulty of nutrient uptake by tumor cells and may also cause spatial heterogeneity in the distribution of nutrients in tumors [6]. During metastasis, flow shear stress in the bloodstream can induce mitochondrial dysfunction and disrupt normal energy metabolism in tumor cells [7]. Finally, the differences in nutrient composition among various tissues can hinder the comprehensive uptake of nutrients by tumor cells, thereby restricting multiple metabolic pathways and leading to energetic stress [8].

In this review, we focus on how different microenvironments impose energetic stresses on tumor cells at various developmental stages and the flexible adaptive mechanisms employed by tumor cells in response. This provides a new perspective for comprehensive research and exploration of therapeutic strategies targeting tumor metabolism.

## Factors Leading to Energy Stress in the Tumor Microenvironment (TME)

### Nutrient shortage

**Figure 1:**
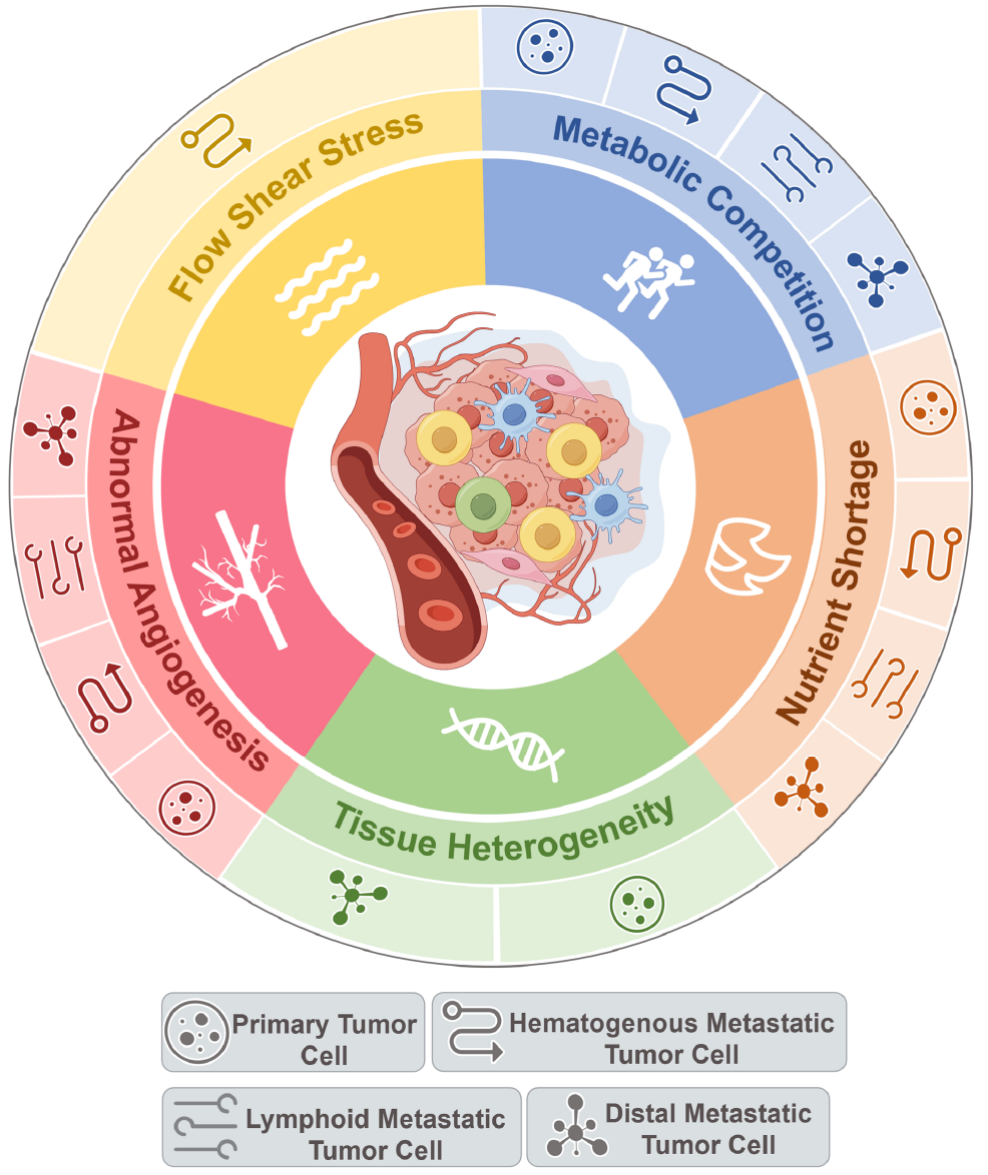
Figure 1 Five major energy stresses that cancer cells encounter throughout their development These include nutrient shortages, metabolic competition, abnormal angiogenesis, fluid shear stress, and tissue heterogeneity. This figure was created via Figdraw (www.figdraw.com).

Energy metabolism reprogramming is a hallmark of cancer. Notably, cancer cells rely on glycolysis for energy production even under oxygen-rich conditions, a phenomenon known as the Warburg effect [9]. In contrast to the 32 molecules of ATP generated through complete glucose oxidation by oxidative phosphorylation, tumor cells produce only two molecules of ATP per glucose molecule consumed during aerobic glycolysis [10]. This metabolic shift intensifies the conflict between high energy demands and the inefficiency of the Warburg effect, leading to nutrient depletion in the TME. Research has shown that oncogenic signals, such as Ras, Myc, and PI3K, can drive the Warburg effect, leading to increased glucose consumption within the tumor microenvironment [11]. Clinical studies have confirmed that glucose levels within the tumor microenvironment are typically three to ten times lower than those in surrounding normal tissues [12]. In addition to glucose, various amino acids, such as glutamine, serine, and arginine, are deficient in the TME [ 13, 14] . Moreover, the capacity of tumor cells to sustain proliferative signaling, evade growth suppression, and resist cell death creates a fundamental conflict between limited resource availability and the cells’ potential for unchecked growth [15]. This challenge is compounded by the inadequate vascularization of tumors, which limits nutrient and oxygen delivery to tumor tissues, exacerbating energy stress [16]. The combination of these factors results in severe nutrient shortages, which persist throughout tumor progression, from primary tumor growth to distant metastasis ( Figure 1).

### Metabolic competition

In normal tissues, tumor cells act as foreign invaders. It can elicit resistance from resident cells and immune cells within the microenvironment, creating metabolic competition. Various activated immune cells—including B cells, T cells, dendritic cells (DCs), and macrophages—serve as competitors, working against tumor occurrence and progression [17]. In addition, cells necessary for normal tissue function, such as endothelial cells and fibroblasts, also compete with tumor cells for limited metabolic resources in the microenvironment. A recent study revealed that myeloid immune cells, especially macrophages, exhibit the highest glucose uptake in the tumor microenvironment, even surpassing T cells and tumor cells [18].

Tumor cells that metastasize to distant organs face additional competition from specialized cells within the target organ microenvironment. In bone tissue, for example, osteoblasts, osteocytes, and osteoclasts, which are involved in bone remodeling, consume large amounts of energy in the microenvironment [19]. Similarly, in the liver, resident cells such as liver sinusoidal endothelial cells (LSECs) rely on energy to maintain normal hepatic function [20]. In the brain, neurons and glial cells continually consume energy to sustain neural activity, increasing metabolic demands [21]. These native cells in distant organs ultimately compete for resources, restricting the energy available to metastatic tumor cells.

Thus, metabolic competition between cancer cells and various resident cells within the tumor microenvironment plays a critical role in energy stress throughout the progression of both primary and metastatic tumors ( Figure 1).

### Abnormal angiogenesis

Blood vessels act as ‘highways’ for nutrient transport throughout the body, serving both normal and tumor cells. However, rapid tumor cell proliferation outpaces the capacity of normal blood vessels, resulting in an inadequate blood supply and restricted nutrient delivery to tumor tissues. In the early stages of tumor development, vascularization is insufficient [22]. This forces tumor cells to rely primarily on tissue infiltration to access external nutrients [22]. As the tumor grows, new blood vessels begin to form. However, the signals that regulate vascularization within tumors are unbalanced [15]. This leads to blood vessels often developing abnormally, resulting in tortuous and disorganized structures with high heterogeneity [ 15, 23] .

Additionally, as tumor cells compete with surrounding host cells for expansion, the abnormal blood vessels within the tumor may become compressed or even collapse, further restricting blood flow [23]. This limitation often restricts tumor size to 2–3 millimeters because of insufficient oxygen and nutrient supplies [ 22, 23] . Moreover, the capillary network within lymph nodes (LNs) is also inadequate to support tumor cells that spread locally [24]. Metastatic tumor cells within LNs are typically located 100–200 μm away from preexisting blood vessels in the surrounding cortex, which aligns with the maximum diffusion range of oxygen [25].

These findings indicate that inadequate vascularization and vascular heterogeneity lead to nutrient deficiencies and hypoxia in tumor tissues. Therefore, tumor cells originating from primary tumors, regional lymph nodes, and distant metastatic sites must employ strategies to overcome these challenges ( Figure 1).

### Fluid shear stress

For metastatic tumor cells, the increased intravascular fluid flow rate presents unique challenges. Fluid shear stress (FSS) generated by high-speed blood flow disrupts mitochondrial function in tumor cells. FSS treatment at 5–30 dyn/cm² has been reported to increase intracellular ROS levels in circulating tumor cells (CTCs). This leads to oxidative stress, mitochondrial dysfunction, and disruptions in normal energy metabolism [7]. Additionally, long transit during metastasis limits metabolic substrates, cytokines, and growth factors, further challenging circulating tumor cells energetically [26].

In contrast, the FSS within lymphatic vessels is approximately 12 dyn/cm²—substantially lower than that in blood vessels due to the shorter length and blind-ended structure of lymphatic channels [27]. This lower FSS is typically insufficient to create significant energy stress on tumor cells migrating via the lymphatic system [27]. Current research suggests that tumor cells experience greater energy stress within the lymph nodes than during their passage through lymphatic vessels [24]. Therefore, the energy stress caused by FSS mainly occurs during the process of tumor metastasis, particularly during hematogenous (blood-borne) dissemination ( Figure 1).

### Tissue heterogeneity in microenvironments

In addition to the previously mentioned factors, it is essential to consider the unique characteristics of different tissue microenvironments where tumor cells reside. Tumors most frequently originate in organs such as the lung, breast, colon, liver, stomach, and prostate [28], whereas common metastatic sites include the lung, liver, bone, and brain [29] ( Figure 1).

Bone tissue, for example, is a dynamic remodeling structure with low oxygen levels that maintains calcium homeostasis through continuous turnover. This “bone circulation” process consumes substantial nutrients, creating local energy shortages that impose significant stress on tumor cells [30]. Moreover, tumor cells colonizing bone must overcome highly hypoxic conditions and rely on the relatively sparse microvascular network in the bone marrow for nutrient transport [31].

In the lungs—an organ directly exposed to the external environment—resident immune cells, such as macrophages, dendritic cells, and a population of memory T cells within the pulmonary airways, contribute to intense competition for resources with metastatic cancer cells [32]. Additionally, the ratio of pyruvate/glutamine in the lung interstitial fluid is threefold greater than that in the general circulation [33]. This appears to limit the diversity of energy sources available to metastatic lung tumor cells to some extent.

Additionally, the liver is the primary site of fructose metabolism [34]. However, tumor cells within liver tissue are unable to directly utilize fructose for energy metabolism, thereby limiting the range of nutrients available to these cells [35]. In the brain, neurons and specialized cells such as astrocytes, oligodendrocytes, and microglia are highly metabolically active, creating a low-energy storage environment within brain tissue [36]. Furthermore, the high lipid content of the brain places additional energy demands on tumor cells, further intensifying metabolic stress [36].

## Adaptation and Counterattack of Primary Tumor Cells under Energy Stress

The primary tumor serves as the origin of tumor progression, where tumor cells must contend with limited energy resources and compete with surrounding normal cells for nutrients. Additionally, insufficient blood vessel distribution often exacerbates energy deprivation in primary tumor cells. The following sections discuss how primary tumor cells adapt to these energy constraints, enabling their survival and promoting tumor growth.

### Exceptional energy competitiveness

#### Enhanced nutrient absorption capability

**Figure 2:**
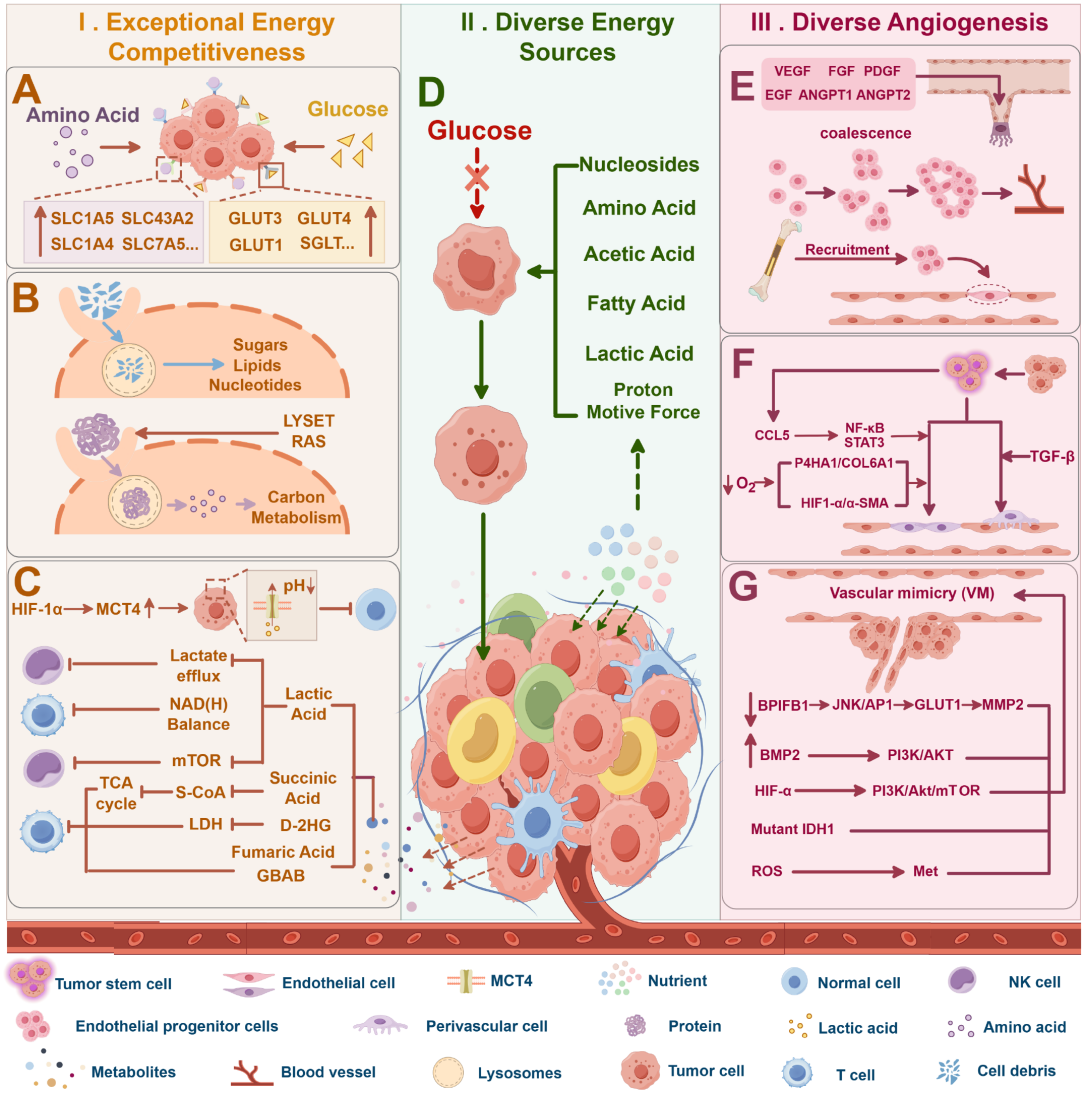
Figure 2 Summary of three main mechanisms by which primary tumor cells resist the energetic stress of the microenvironment I. Exceptional energy competitiveness: (A) enhanced nutrient absorption capability, (B) macropinocytosis, and (C) multiple metabolites inhibit the activity of other cells. II. Diverse energy sources (D). III. Diverse angiogenesis mechanisms: (E) endothelial cell source, (F) tumor stem cell source, and (G) tumor cell source. This figure was created via Figdraw (www.figdraw.com).

Cancer cells can significantly increase their nutrient uptake rate, thereby gaining a competitive advantage in terms of energy acquisition. Indeed, cancer cells consume approximately two-thirds of the glucose available in the microenvironment, with the remaining one-third used by bone marrow cells, such as B cells and T cells, and other cell types [18]. The robust glucose uptake capacity of cancer cells is closely related to the expression levels of glucose transport proteins on their membranes [37]. For example, sodium-independent glucose transporter 1 (GLUT1) is notably upregulated in cancers such as hepatocellular carcinoma, pancreatic tumors, and prostate cancer [37]. GLUT2 is overexpressed in hepatocellular carcinoma and colorectal cancer, whereas GLUT3 is elevated in papillary thyroid carcinoma and oral squamous cell carcinoma [37]. Additionally, GLUT4, GLUT5, GLUT6, and GLUT12 are upregulated in most cancer types [37]. Cancer cells also increase glucose uptake by increasing sodium-dependent glucose co-transporter (SGLT) expression [38]. While glucose is the primary substrate for GLUTs, these transporters also facilitate the transport of other substrates, such as fructose, galactose, and mannose [39] ( Figure 2A).

In addition to glucose, compared with normal cells, tumor cells also exhibit increased amino acid uptake. Tumor cells overexpress glutamine transporters such as SLC1A5, SLC38A1, SLC38A2, and SLC38A5 to facilitate glutamine uptake [40] ( Figure 2A). Furthermore, the serine transporter SLC1A4 and the branched-chain amino acid transporter SLC7A5 are upregulated in lung and breast cancers [40] ( Figure 2A). Additionally, tumor cells can compete with T cells for methionine by overexpressing the methionine transporter SLC43A2, thereby impairing T cell function [41].

#### Macropinocytosis

Macropinocytosis is a highly conserved endocytic process that enables cells to internalize extracellular fluid and its contents through large, heterogeneous vesicles [42]. This process is an essential nutrient acquisition pathway for tumor cells, providing a significant source of amino acids [43]. Targeting macropinocytosis has been shown to effectively inhibit tumor growth [44]. Extracellular proteins, taken up via macropinocytosis, are hydrolyzed in lysosomes to generate amino acids such as glutamine, which then fuel cancer cell metabolism [45].

RAS-transformed cancer cells can harness macropinosomes to internalize extracellular proteins, supplying themselves with amino acids [46]. Indeed, pancreatic ductal adenocarcinoma (PDAC) cells can absorb extracellular matrix (ECM) proteins, such as type I and IV collagen, through macropinocytosis driven by mutant K-RAS [47]. Moreover, the lysosomal enzyme trafficking factor (LYSET, also known as TMEM251) is indispensable for cancer cell survival when it utilizes extracellular proteins via macropinocytosis [48]. LYSET-deficient cancer cells cannot thrive in amino acid-deprived environments [48]. In addition, cancer cells also uptake necrotic debris via macropinocytosis, repurposing sugars, lipids, and nucleotides as nutrients under scarcity, increasing metabolic adaptability [49] ( Figure 2B).

#### Multiple metabolites inhibit the activity of other cells

Monocarboxylate transporters (MCTs) on the cellular membrane are key proteins for lactate transport [50]. In hypoxic areas distant from blood vessels, HIF-1α activation significantly upregulates MCT4 to facilitate lactate export [51]. This acidity impedes lactate efflux in nontumor cells, causing intracellular acidosis and potentially leading to reduced activity or apoptosis [52]. Studies have shown that the accumulation of tumor-derived lactate in the microenvironment can inhibit the excretion of lactate by NK cells, reduce the intracellular pH, and induce mitochondrial dysfunction and apoptosis [53]. In addition, lactic acid affects the balance of the NAD(H) redox status of T cells, affecting the normal metabolic processes of T cells [54]. Furthermore, lactic acid can inhibit the expressions of IFN-γ and interleukin-4 in NKT cells in the microenvironment by blocking the conduction of the mTOR signaling pathway and inhibiting the activation of immune cells such as NK cells [55].

In addition to lactic acid accumulation, the accumulation of tumor-derived fumaric acid in the microenvironment disrupts normal metabolic processes [56]. Furthermore, tumor cell-derived succinic acid downregulates succinyl-CoA, blocking the normal TCA cycle and thereby inhibiting energy metabolism in T cells [57]. Moreover, tumor-derived γ-aminobutyric acid (GABA) can hinder T-cell infiltration into the tumor microenvironment and can also directly inhibit the activation of T cells [58]. Additionally, D-2-hydroxyglutarate (D-2HG), a metabolite that accumulates in the microenvironment due to IDH mutations in cancer cells, directly inhibits lactate dehydrogenase in T cells, disrupting their normal glucose metabolism [59] ( Figure 2C).

In addition to tumor cells, tumor-associated macrophages (TAMs) and cancer-associated fibroblasts (CAFs) significantly impact the tumor microenvironment. TAMs secrete itaconic acid, causing T-cell depletion and reduced proliferation/activation [60]. In addition, prostaglandin E2 (PGE2) secreted by CAFs has been found to inhibit the function of T cells in lung cancer and NK cells in colon cancer [61]. Moreover, lactate secreted by CAFs prevents the differentiation, activation, and antigen presentation of monocytes, MDSCs, and dendritic cells (DCs) [62].

These findings suggest that cancer cells can inhibit the metabolic activities of neighboring cells by secreting specific metabolites, thereby reinforcing their competitive advantage ( Figure 2C).

### Diverse energy sources

Tumor cells can draw on a variety of energy sources beyond glucose, including amino acids, lipids, nucleotides, lactate, acetate, and proton motive force ( Figure 2D). This metabolic flexibility allows tumor cells to withstand energy stress and sustain growth under nutrient-limited conditions, highlighting their capacity for metabolic adaptation.

#### Fatty acids

Dysregulated lipid metabolism, particularly abnormal fatty acid metabolism, is a critical feature of metabolic reprogramming in cancer cells. Under glucose deprivation, ERK2 activation triggers Nur77 translocation to the mitochondria, where it protects TP-β from oxidative inactivation, thereby promoting fatty acid oxidation (FAO) and supporting tumor cell survival [63]. Additionally, under energy deficiency, choline kinase alpha 2 (CHKα2) undergoes sequential phosphorylation by AMPK and acetylation by KAT5, leading to its translocation from the cytosol to the surface of lipid droplets [64]. CHKα2 phosphorylates the surface proteins PLIN2/3 of lipid droplets, which are then recognized by the molecular chaperone HSC70 and degraded via the autophagy pathway, exposing lipid molecules [64]. These lipids, which are subsequently broken down by lipases and autophagosomes, serve as energy sources that facilitate tumor progression [64]. Further research revealed that glucose starvation promotes the interaction between PFKP and AMPK. This enhances AMPK recruitment to mitochondria, leading to ACC2 phosphorylation and increased oxidation of long-chain fatty acids to maintain energy and redox balance [65].

#### Amino acids

Abnormalities in amino acid metabolism, particularly glutamine metabolism, play essential and multifaceted roles in supporting the survival of various cancer cells [40]. Many cancer cells depend heavily on glutamine breakdown to replenish intermediates in the TCA cycle [66]. In glucose-deprived environments, glutamine metabolism significantly increases the production of citric acid, malic acid, and fumaric acid, driving a TCA cycle that does not rely on glucose [67]. It has been reported that cancer cells upregulate malic enzyme 1 (ME1) during glucose starvation to increase glutamine utilization [68]. Recently, our group showed that glucose starvation upregulates mitochondrial MT-CO2 expression, which facilitates glutaminolysis and tumor cell survival [69]. Additionally, glucose deprivation activates the AMPK/PDZD8/GLS1 pathway, which promotes glutamine breakdown and helps sustain cancer cell survival [70]. Under hypoxic conditions, hypoxia-inducible factor (HIF) activation accelerates glutamine metabolism in cancer cells by increasing glutaminase 1 (GLS1) expression [71]. Furthermore, glutamine can upregulate GLS and glutamate dehydrogenase (GDH) activity, thereby enhancing the catabolic metabolism of cancer cells [72].

In the absence of glutamine, brain tumor cells can utilize aspartate as an alternative to glutamine for proliferation [73]. Additionally, under glucose deprivation, leucyl-tRNA synthetase 1 (LARS1) undergoes phosphorylation, which reduces its binding to leucine, leading to increased levels of free leucine that aid in maintaining cancer cell survival [74].

#### Lactic acid

Lactic acid has traditionally been viewed as a byproduct of glycolysis, but recent research has indicated that lactate can be utilized as a metabolic substrate to enter the TCA cycle and produce energy [55]. Lactate concentrations in tumor tissue are more than ten times greater than those in normal tissue [51]. Isotope labeling analyses revealed that more than 50% of the TCA cycle intermediates in glucose-restricted cancer cells are derived from lactate [75]. Another study indicated that cancer cells can use lactate to produce NADPH with the assistance of isocitrate dehydrogenase 1 (IDH1) under glucose starvation conditions [68]. Additionally, in cancer cells, lactate entering the mitochondria is not only oxidized by lactate dehydrogenase B (LDHB) to form pyruvate for the TCA cycle but also serves as a carbon source for lipid synthesis [76]. These findings indicate that lactate can support cancer cell survival under glucose-restricted conditions.

#### Others

Cancer cells can also obtain energy from nucleosides, acetic acid, and the proton motive force. Recent studies have shown that at least three types of nucleosides, such as uridine, hypoxanthine nucleoside, and thymidine, can be utilized by cancer cells upon glucose deprivation [ 77– 79] . Notably, uridine can contribute to energy metabolism in cancer cells even in the presence of glucose [77]. Guanosine also enhances cell survival under glucose deprivation conditions by increasing Rag GTPase levels and promoting the synthesis of enzymes associated with the TCA cycle [80].

Acetic acid can combine with coenzyme A to form acetyl-CoA upon energy deprivation, which fuels the TCA cycle for energy production [81]. Moreover, cancer cells can synthesize ATP via proton gradients [82]. The pH of cancer cells is relatively alkaline [82]. This allows them to obtain energy through intracellular and extracellular pH differences (extracellular acidity, intracellular alkalinity), a mechanism that has not been observed in normal cells [83].

### Diverse angiogenesis

Tumor cells counteract energy stress through diverse angiogenesis mechanisms, primarily involving endothelial cells (ECs), cancer stem cells (CSCs), and tumor cells themselves [ 84– 86] . ECs drive neovascularization via a three-step process—tip cell selection, bud extension, and lumen formation—which is mediated by vascular endothelial growth factor (VEGF) [84] ( Figure 2E). VEGF directly stimulates EC proliferation and movement and indirectly activates matrix metalloproteinases (MMPs) and extracellular signal-regulated kinases (ERKs) [ 87, 88] . Other factors, such as PDGF, EGF, and FGF, enhance angiogenesis by upregulating VEGF or inducing EC-derived vessels [ 89, 90] . However, cytokines ( *e.g.*, CCL and CXCL12) recruit endothelial progenitor cells (EPCs) to the tumor microenvironment [ 91, 92] .

Additionally, CSCs can differentiate into endothelial or smooth muscle-like cells to support neovascularization [85] ( Figure 2F). For example, glioblastoma stem-like cells (GSCs) can undergo endothelial differentiation via the P4HA1/COL6A1 axis under hypoxia [93]. Alternatively, they can be recruited by ECs via the SDF-1/CXCR4 axis and then induced by TGF-β to form perivascular cells [94]. Breast and ovarian cancer stem cells also promote angiogenesis through the HIF-1α/α-SMA or NF-κB/STAT3 pathways [ 95, 96] .

Tumor cells can also form vascular-like structures through vascular mimicry (VM) by secreting collagen, proteoglycans, and laminin [86] ( Figure 2G). Mitochondrial ROS activate Met to promote VM in melanoma, whereas HIF-α drives VM via VEGF-α secretion and the PI3K/Akt/mTOR pathway [ 97, 98] . RUNX1 promotes BMP2 expression to induce VM in laryngeal squamous cell carcinoma (LSCC) [99]. Mutating IDH1 in glioma cells also facilitates VM [100]. Furthermore, the downregulation of BPIFB1 further supports VM formation by activating the JNK/AP1 pathway and enhancing histone acetylation [101].

## Mechanisms of Energy Stress Adaptation in Metastasizing Cancer Cells

Currently, it is widely accepted that cancer cells metastasize primarily through hematogenous and lymphatic spread [102]. Circulating tumor cells (CTCs) within blood vessels face significant challenges posed by FSS, whereas cancer cells that spread via the lymphatic system must adapt to the distinctive microenvironment within LNs.

### Hematogenous metastasis

#### Diverse options in transfer strategies

**Figure 3:**
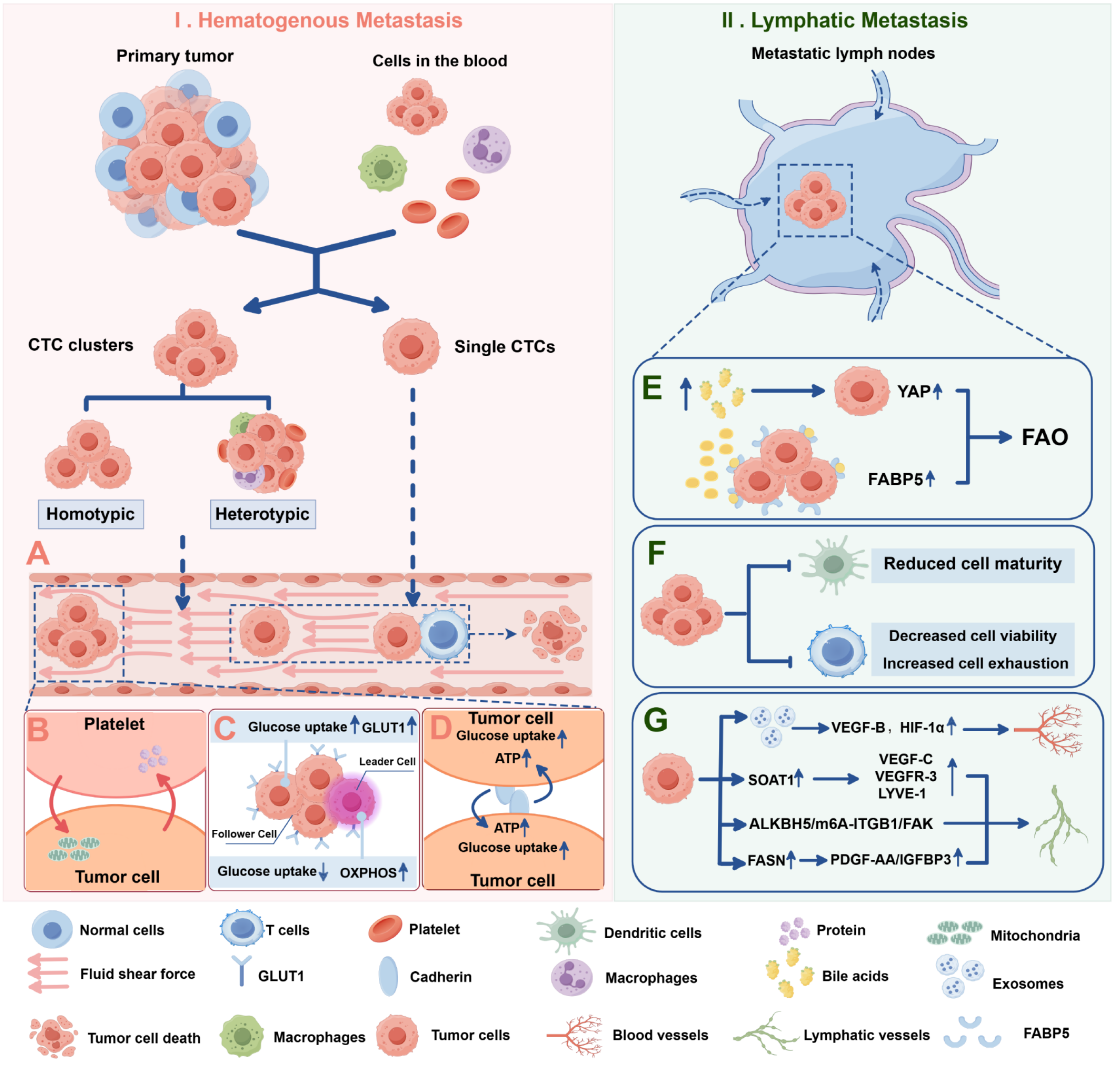
Figure 3 Metastatic tumor cells display distinct responses to energy stress in the lymphatic and hematologic systems I. Hematogenous metastasis: (A) tumor cells form circulating tumor cell (CTC) clusters to resist fluid shear forces; (B) tumor cells exchange intracellular substances with platelets to increase their metabolic capacity; (C) circulating tumor cell (CTC) clusters can be subdivided into leader and follower cells, which adopt distinct metabolic programs to cope with energy stress; (D) cadherin-mediated mechanical coupling promotes glucose uptake and ATP production in tumor cells. II. Lymphatic metastasis: (E) tumor cells increase fatty acid metabolism to counteract energy stress in the lymph node. (F) Lymphatic tumor cells inhibit the normal function of nontumor cells. (G) Lymphatic metastatic tumor cells secrete exosomes and cytokines to promote lymphatic vessel growth and angiogenesis. This figure was created via Figdraw (www.figdraw.com).

The metastasis of tumor cells mainly depends on single-cell migration and collective migration [103]. Collective metastasis mainly occurs in the form of CTC clusters and can form homotypic CTC clusters and heterotypic CTC clusters through collective and aggregation methods [104]. In addition, there is much evidence that CTC clusters are better able to resist FSS and cope with energy stress than single CTC [103] ( Figure 3A).

Interestingly, while collective cell migration in the form of cell clusters appears to be more energy intensive from an energy stress perspective, it is more common in metastatic cells than in individual tumor cells [ 105, 106] . On the one hand, collective migration can use energy more efficiently and respond more flexibly to energy stress [106]. The cells in the CTC cluster can be divided into leader cells and follower cells [107]. Studies have shown that leader cells preferentially use mitochondrial respiration for energy production, resulting in increased OXPHOS dependence and decreased glucose uptake [108] ( Figure 3C). Subsequent cells rely on glycolysis to meet energy requirements, resulting in increased GLUT1 expression and glucose uptake [108]. Surprisingly, when the energy of the leader cell is depleted, the leader cell transforms into a follower cell and creates a new leader cell [106]. The division of labor in CTC clusters creates an energy preference among tumor cells. This allows CTC clusters to use nutrients more efficiently than single cells do during energy-limited migration, thus resisting energy stress. Indeed, when the energy cost of metastasis increases and metastasis becomes difficult, cancer cells move from an individual invasion pattern to a collective invasion pattern [106]. Furthermore, cadherin is required for cell-to-cell adhesion in CTC clusters. The mechanical coupling of cadherin activates metabolic signaling and energy production, promoting glucose uptake and ATP production in tumor cells [105] ( Figure 3D).

#### Survival of cancer cells under fluid shear stress

FSS can be categorized into four levels: micro (0–0.5 dyn/cm²), low (0.5–15 dyn/cm²), medium (15–30 dyn/cm²), and high (> 30 dyn/cm²) [27]. The magnitude of the FSS in human blood vessels typically ranges from 4 to 30 dyn/cm² [109]. This can increase ROS levels within cancer cells, leading to oxidative stress and mitochondrial dysfunction. However, CTCs can bind to platelets, forming platelet-tumor cell complexes to reduce FSS-mediated ROS and prevent mitochondrial dysfunction [110]. The main mechanisms by which tumor cells bind to platelets include: (1) direct contact, such as podoplanin on the tumor cell membrane binding to the activated receptor CLEC-2 on platelets [111]; (2) the release of platelet agonists triggering coagulation reactions, where tumor cells secrete tissue factor (TF) to mimic vascular injury and activate platelets via the extrinsic coagulation pathway [112]; and (3) indirect activation of platelets through immune cells, such as neutrophils forming platelet-neutrophil-tumor cell complexes under the influence of cancer mucin and selectins and releasing cathepsin G to further promote platelet activation and aggregation [113]. Interestingly, tumor cells can also acquire mitochondria from platelets to increase their metastatic potential [114] ( Figure 3B). Additionally, the protein composition of tumor cell-associated platelets during bloodstream metastasis changes [115]. This is partially due to the absorption of proteins from cancer cells, which enable platelets to support tumor cell metastasis [115]. These observations suggest that platelets and cancer cells may maintain normal energy metabolism by exchanging proteins, glucose, and other intracellular nutrients, thereby supporting tumor cell survival under FSS.

### Lymphatic metastasis

Lymphatic metastasis refers mainly to the process by which cancer cells spread to other organs through lymphatic vessels to LNs as the main channel [24]. It has been reported that bile acid levels that accumulate in metastatic LNs are greater than those in other tissues [116]. In this unique microenvironment, characterized by the nutrient composition of LNs, tumor cells gradually adapt to rely on energy produced through fatty acid metabolism to overcome energy stress. Bile acids that accumulate in LNs selectively activate Yes-associated protein (YAP), promoting FAO in tumor cells [116]. Pharmacological inhibition of fatty acid oxidation or knockout of *YAP* in cancer cells inhibits lymph node metastasis but not hematogenous metastasis [117]. Fatty acid binding protein 5 (FABP5) has also been found to play a role in reprogramming fatty acid metabolism, thereby facilitating tumor cell metastasis to lymph nodes [118]. These findings highlight the importance of fatty acid metabolism as a critical strategy for tumor cells to counteract energy stress in the lymph node microenvironment ( Figure 3E).

Additionally, clinical data have shown a decrease in the proportion of mature dendritic cells in metastatic lymph nodes in melanoma and breast cancer patients [119]. Furthermore, single-cell RNA sequencing of sentinel lymph nodes in patients with melanoma and head and neck squamous cell carcinoma revealed that CD8 ^+^ T cells exhibit reduced activation and function in metastatic lymph nodes [ 120, 121] . These findings indicate that cancer cells in LNs can adapt to metabolic competition by reducing immune cell numbers, impairing immune cell functions, and thereby decreasing their metabolic demands ( Figure 3F).

Relying solely on the preexisting vascular distribution around lymph nodes to support the energy needs of cancer cells is often insufficient. It has been shown that tumor cells release exosomes to interact with various cells within the LNs [122]. This induces increases in VEGF-B, HIF-1α, and multiple proangiogenic factors in the LN microenvironment, which promote the formation of the tumor vasculature [122]. Notably, high SOAT1 expression in metastatic cancer cells within LNs is positively correlated with the expression of lymphangiogenic factors such as VEGF-C, VEGFR-3, and LYVE-11 [123]. Moreover, tumor cells can enhance tumor-associated lymphangiogenesis via the ALKBH5/m6A-ITGB1/FAK signaling axis [124]. In addition, tumor cells can upregulate the secretion of PDGF-AA/IGFBP3 through the oncogenic factor fatty acid synthase (FASN), promoting the formation of tumor-associated lymphatic vessels [125] ( Figure 3G).

These observations demonstrate that tumor-associated lymphatic vessels play an important role in the development of tumor tissue. It not only provides an additional pathway for tumor cells to metastasize to lymph nodes but also improves the efficiency of nutrient transport.

## Mechanisms of Cancer Cell Adaptation to Energy Stress at Distant Metastatic Sites

**Figure 4:**
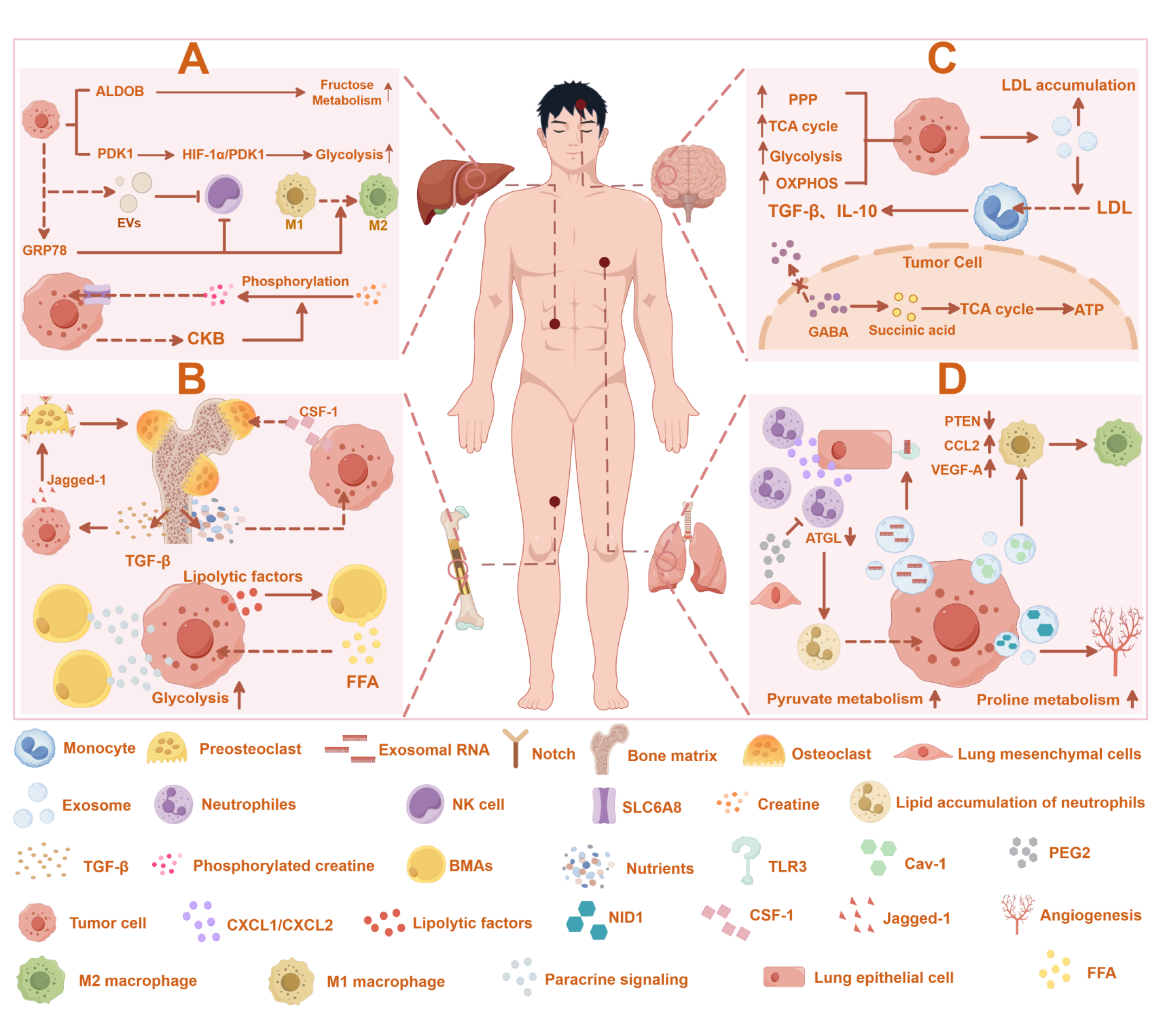
Figure 4 Adaptation mechanisms of metastatic tumor cells to energy stress (A) Liver metastatic tumor cells adapt to energy stress via metabolic reprogramming and form premetastatic niches. (B) Bone metastatic tumor cells utilize bone matrix nutrients, recruit osteoclasts, and repurpose bone cells to adapt to energy stress. Brain (C) or lung (D) metastatic tumor cells meet energy demands via metabolic reprogramming and create an immunosuppressive niche. This figure was created via Figdraw (www.figdraw.com).

Dormancy is a strategy employed by various tumor cells to cope with energy stress within the microenvironment of distant metastases [126]. When tumor cells are unable to adapt actively to energy stress through metabolic reprogramming, they may enter a dormant state characterized by low metabolism, slow proliferation, and halted reproductive growth [127]. However, when the external environment changes, dormant tumor cells can be reactivated and adopt more aggressive strategies to manage energy stress [127]. For example, persistent inflammation can stimulate lung-resident neutrophils to secrete extracellular traps (NETs), which can reawaken dormant cancer cells [128]. Similarly, the process of bone remodeling, driven by osteoclasts in bone tissue, can also trigger the activation of dormant cancer cells [129]. We propose that the primary mechanisms by which tumor cells resist energy stress during distant metastasis include the formation of pre-metastatic niches and metabolic adaptation. Additionally, owing to the substantial tissue heterogeneity of distant metastatic sites, the adaptive strategies employed by tumor cells with different tissue-specific metastatic potentials are diverse ( Figure 4).

### Liver

The liver is particularly prone to tumor metastasis. Metastatic colon cancer cells in the liver have been shown to upregulate aldolase B (ALDOB), reprogramming their metabolism to increase fructose utilization [130]. Furthermore, in the hypoxic microenvironment of the liver, colon cancer cells phosphorylate extracellular creatine via creatine kinase B (CKB) and utilize the SLC6A8 transporter to absorb phosphocreatine for ATP production [130]. Moreover, compared with their primary counterparts, liver metastatic breast cancer cells overexpress PDK1 and amplify glycolytic metabolism through the HIF-1α/PDK1 signaling axis [131]. However, there is currently no evidence indicating that liver metastatic tumor cells utilize the same metabolic pathways to manage starvation stress ( Figure 4A).

Tumor-derived extracellular vesicles (EVs) have been shown to facilitate the formation of liver pre-metastatic niches characterized by NK cell dysregulation [132]. The ability of NK cells to uptake glucose is down-regulated, thereby reducing metabolic stress in tumor cells [132]. Moreover, tumor-secreted GRP78 recruits macrophages and induces their M2 polarization while promoting increased TGF-β levels in the liver microenvironment to construct a pre-metastatic niche that minimizes energy stress on cancer cells [133] ( Figure 4A).

### Bone

Bone metastatic tumor cells often establish themselves in distinct microenvironments, with some settling near blood vessels to form perivascular niches, whereas others create endosteal niches [134]. In niches that are distant from blood vessels, tumor cells exploit the bone matrix as a vital “energy refueling station” [134]. Osteolytic bone metastasis is a key process in the formation of tumor bone metastases [134]. Tumor cell invasion recruits osteoclasts and stimulates their development and activation by secreting CSF-1, leading to increased local bone matrix degradation [135]. This process releases essential nutrients, minerals, and growth factors from the bone matrix [134]. Notably, the release of TGF-β during bone matrix degradation can induce Jagged-1 expression in tumor cells. Increased Jagged-1 binds to Notch receptors on pre-osteoclasts to promote osteoclast generation and accelerate bone matrix breakdown, thereby creating a self-perpetuating cycle that supports cancer cell survival and proliferation [136]. Moreover, bone cells, the most abundant cells in bone, can be re-edited by cancer cells to create a microenvironment that favors bone resorption rather than bone formation [137].

Bone marrow adipocytes (BMAs) are essential components of bone tissue and play a significant role in supporting tumor cell survival [138]. BMAs can induce HIF-1α activation through a “pseudo-hypoxic” mechanism similar to that induced by lactate, thereby enhancing the glycolytic phenotype of tumor cells [138]. Additionally, tumor cells can stimulate the breakdown of lipids in BMAs into free fatty acids (FFAs) via lipidolytic factors, providing an energy source for their metabolic needs [139] ( Figure 4B).

Although bone metastasis is common in the progression of many tumors, both basic research and clinical data indicate that certain bones, such as those in the hands or feet, are less conducive to tumor cell colonization [140]. The underlying reasons for this selective phenomenon, however, remain unclear.

### Brain

Brain metastatic tumor cells have been reported to upregulate the expressions of metabolic enzymes linked to glycolysis, the TCA cycle, OXPHOS, the pentose phosphate pathway (PPP), and the glutathione system [141]. This metabolic adaptation enables cancer cells to meet energy demands in brain tissue, which inherently has low energy storage. While brain tissue is lipid rich, the availability of these lipids is limited [142]. This prompts cancer cells to exploit them through recycling and re-synthesis pathways to sustain metabolic needs [142]. Notably, brain-metastatic tumor cells can mimic brain-specific metabolic traits. For example, GABA is converted into succinate to fuel the TCA cycle and increase ATP production [143]. Furthermore, EVs derived from brain metastases facilitate LDL accumulation and EV uptake by monocytes, triggering the secretion of immunosuppressive factors, including TGF-β and IL-10, to establish pre-metastatic niches in brain tissue [144] ( Figure 4C).

### Lung

Pulmonary metastasis is common in the advanced stages of many cancer types [145]. Lung metastatic tumor cells have been shown to secrete exosomal RNA [146]. This RNA activates Toll-like receptor 3 (TLR3) in pulmonary epithelial cells, creating an immunosuppressive pre-metastatic niche characterized by neutrophil infiltration [146]. A recent study indicated that pulmonary mesenchymal cells can inhibit the activity of adipose triglyceride lipase (ATGL) in neutrophils through both PGE2-dependent and PGE2-independent mechanisms, resulting in the accumulation of neutral lipids in infiltrating neutrophils [147]. Lung metastatic tumor cells exploit these accumulated lipids via macropinocytosis-lysosomal pathways as an energy source to support their growth [147]. Furthermore, compared with primary tumor cells, tumor cells colonizing the lungs significantly rely on pyruvate metabolism and utilize pyruvate at a greater rate [148]. In addition, the proline cycle, including catabolism by proline dehydrogenase (PRODH) and anabolism by pyrroline-5-carboxylate reductase 1 (PYCR1), is critical for tumor lung metastasis [149] ( Figure 4D).

Tumor cells can increase Cav-1 levels in the pulmonary microenvironment by secreting exosomes, which subsequently activate the PTEN/CCL2/VEGF-A pathway [150]. This promotes M2-type macrophage polarization and angiogenesis, enabling tumor cells to better manage energy stress [150]. Additionally, tumor-derived extracellular vesicles can transport nidogen-1 (NID1) to lung tissue [151]. This enhances endothelial permeability and angiogenesis, thereby creating a premetastatic niche that supports the metabolic needs of tumor cells [151] ( Figure 4D).

These findings highlight the distinct metabolic adaptation mechanisms of tumor cells in different metastatic organs. However, some metastases exhibit metabolic profiles highly similar to those of the primary tumor. Metastatic tumor cells in pancreatic, lung, and liver cancers display remarkably similar patterns of glucose and glutamine metabolism compared with those in primary tumors [152]. These findings suggest that metastatic cells may preferentially target tissues with metabolic features resembling those of the primary site, facilitating their adaptation and enhancing their capacity to endure energy stress in the metastatic niche.

## Targeting Metabolism in Tumor Therapy

The insights discussed above underscore that targeting tumor metabolism through strategies such as depleting energy sources, inhibiting angiogenesis, and disrupting key metabolic pathways are pivotal for suppressing cancer progression. Here, we outline the current practical applications of targeted metabolic therapies in oncology.

Extensive research on anticancer drugs that target energy metabolism has demonstrated that single-drug therapies often lead to tumor cell resistance [153]. For tumor cells capable of utilizing multiple adaptable energy sources, single-drug treatments do not appear to be sufficient [ 153, 154] . Enhancing anticancer efficacy therefore requires novel treatment strategies that combine drugs targeting different metabolic pathways of the same energy source or drugs targeting distinct energy sources.

The combination of CB-839 and 2-PMPA, which target distinct glutamine metabolic pathways, has been reported to significantly improve therapeutic outcomes compared to monotherapy with either drug alone [155]. Additionally, co-administration of the glutamine metabolism inhibitor BPTES-NPs with the glucose metabolism inhibitor metformin has shown notable therapeutic benefits [155]. Furthermore, the combined use of orlistat, lonidamine, and 6-diazo-5-oxo-L-norleucine (DON) to simultaneously inhibit glycolysis, glutaminolysis, and fatty acid synthesis in colorectal cancer has significant therapeutic effects without adverse effects [156].

On the basis of the characteristics of tumor cells under microenvironmental energy stress discussed above, we compiled a list of anticancer drugs that can induce energy stress in cancer cells ( Table 1). Most of these drugs are in clinical trial phases, and some non-traditional drugs with anticancer potential are also included. For example, recent studies have shown that orlistat, a commonly used weight loss medication, inhibits lipid metabolism in cancer cells, induces energy stress, and suppresses tumor development [156]. Notably, aspirin and warfarin prevent platelet recruitment by CTCs, whereas digoxin disrupts CTC formation, increasing the susceptibility of metastasizing tumor cells to mitochondrial damage induced by FSS [ 163– 165] . Additionally, clodronate and etidronate disrupt mitochondrial function in osteoclasts, inducing apoptosis and subsequently inhibiting bone matrix degradation. This alteration in the bone microenvironment affects the nutritional landscape, thereby influencing the metabolic activities of tumor cells [166].

**Table:** **Table1** A list of anticancer agents associated with the induction of cellular energy stress

Drug	Effect	Cancer	Stage	Ref.
Nintedanib	Block the activity of VEGFR, PDGFR, and FGFR to inhibit angiogenesis	Malignant pleural mesothelioma and prostate cancer	Phase IV clinical	[157]
Everolimus	Inhibit mTOR to suppress the blood vessel mimicry	Neuroendocrine tumors and breast cancer *etc*.	Phase II clinical	[158]
Thalidomide and Lenalidomide	Target VEGF and bFGF to inhibit angiogenesis	Multiple myeloma	Phase III clinical	[159]
Orlistat	Inhibit the lipid metabolism of cancer cells	Colorectal cancer	Preclinical trial	[156]
Lonidamine	Inhibit the breakdown of glutamine by cancer cells	Colorectal cancer	Phase III clinical	[156]
Fluvastatin sodium (Flu)	Inhibit MCT4 and block lactic acid outflow, leading to acidosis and weakening the metabolic competitiveness of cancer cells	A series of human tumor cell lines	Phase II clinical	[160]
Ritonavir	Inhibit GLUT4 to block the absorption and utilization of glucose by cancer cells	Melanoma, myeloma *etc*.	Phase I/II clinical	[161]
Lpilimumab	Promote T cell glycolysis by inhibiting CTLA-4 and weaken the metabolic competitive advantage of cancer cells	Melanoma	Phase IV clinical	[162]
Aspirin and LMWH	Prevent the formation of CTC-platelet clusters to promote FSS-induced energy stress on tumor cells	Primary osteosarcoma, soft tissue sarcoma *etc*.	Phase IV clinical	[ 163, 164]
Digoxin	Destroy the CTC cluster to promote FSS-induced energy stress on tumor cells	Breast cancer	Phase I clinical	[165]
Avasimibe	Inhibit lymphangiogenesis and lipid metabolism of cancer cells in lymph nodes	Gastric carcinoma	Preclinical trial	[123]

However, whether in the preclinical, clinical trial, or clinical use stage, most anticancer drugs currently target the metabolic responses of tumor cells at a single developmental stage. Moreover, the efficacy of these treatments is seldom evaluated comprehensively across the three continuous stages of tumor progression—initiating at the primary site, advancing through metastasis, and culminating in distal colonization.

## Conclusions and Perspectives

Energy stress is a critical factor in tumor progression, influencing the survival, growth, metastasis, and colonization of tumor cells. As tumors evolve, they encounter energy stress within diverse microenvironments, each of which presents distinct challenges. The evidence reviewed here suggests that tumor cells employ adaptive and confrontational strategies to cope with these stresses. On the one hand, tumor cells reprogram their metabolism to adapt to the energy demands of different microenvironments. On the other hand, they alter the microenvironment itself to support their growth and survival. This dynamic interaction between tumor cells and their surroundings is central to our understanding of cancer biology. This finding has profound implications for therapeutic strategies.

Tumor cells experience varying forms of energy stress at different stages of tumor progression, with shifts in preferred energy sources in response to microenvironmental changes. We propose that “precision therapy” tailored to specific stages of tumor development and metastatic sites may more effectively target the energy stress pathways that tumors exploit. For example, in early liver tumor development, drugs targeting the fructose-lipid transition process in the liver may be advantageous [35]. However, in late-stage brain metastasis of liver tumors, lipid metabolism inhibitors such as orlistat may be more effective [153]. For circulating cancer cells, drugs that disrupt interactions between tumor cells and platelets could amplify fluid shear stress effects and impair metabolic function. Targeting fatty acid or lactate metabolism may improve outcomes for patients with lymph node metastases. Additionally, biopsies and metabolic enzyme expression analysis in tumor tissues could enable precise targeting of highly expressed metabolic pathways, limiting cancer cells’ access to preferred energy sources and enhancing therapeutic efficacy.

The metabolic preferences of cancer cells are influenced not only by the availability of nutrients but also by the composition of the plasma and the broader systemic environment [167]. In this context, modifying a patient’s diet to alter the nutritional landscape may complement pharmacological therapies and further support “precision therapy”. Recent studies have confirmed that caloric restriction and intermittent fasting can improve the efficacy and prognosis of various cancers, including breast, lung, and brain cancer [ 168, 169] . Moreover, reducing dietary fructose intake has been shown to significantly inhibit the growth of liver metastatic tumor cells, with minimal effects on primary tumors [130]. Furthermore, omega-3 fatty acid metabolites, once taken up by tumor cells, have been shown to inhibit angiogenesis and exert anticancer effects [170]. These findings suggest that increasing the dietary intake of deep-sea fish may improve treatment outcomes for patients with liver cancer, brain cancer, and other cancers that preferentially utilize fatty acids. Dietary interventions thus represent a promising adjunct to cancer treatment. When designing therapies, it is essential to consider both the diverse energy sources that tumor cells utilize and the heterogeneity of cancer tissues. Adjusting the ratios of carbohydrates, proteins, and lipids in the diets of patients at different treatment stages could enable precise metabolic inhibition, thereby enhancing overall therapeutic efficacy.

Finally, investigating the metabolic shifts that occur during tumor progression requires a comprehensive, panoramic approach. Current animal models and research techniques, however, do not fully capture the complexities of metabolic changes across different stages and cancer types. To gain a deeper understanding of the metabolic landscape of cancer, more sophisticated models that recapitulate metastatic primary tumors across various cancer types are needed. Additionally, real-time monitoring techniques are essential for tracking tumor progression. Such approaches will be essential for elucidating the metabolic dynamics that drive tumorigenesis and metastasis and for the future development of targeted therapies aimed at disrupting these processes.

## References

[1] Zhang S, Xiao X, Yi Y, Wang X, Zhu L, Shen Y, Lin D (2024). Tumor initiation and early tumorigenesis: molecular mechanisms and interventional targets. Sig Transduct Target Ther.

[2] Gerstberger S, Jiang Q, Ganesh K (2023). Metastasis. Cell.

[3] Valastyan S, Weinberg RA (2011). Tumor metastasis: molecular insights and evolving paradigms. Cell.

[4] Pavlova NN, Zhu J, Thompson CB (2022). The hallmarks of cancer metabolism: still emerging. Cell Metab.

[5] Wang B, Pei J, Xu S, Liu J, Yu J (2024). A glutamine tug-of-war between cancer and immune cells: recent advances in unraveling the ongoing battle. J Exp Clin Cancer Res.

[6] Demicco M, Liu XZ, Leithner K, Fendt SM (2024). Metabolic heterogeneity in cancer. Nat Metab.

[7] Fu A, Ma S, Wei N, Xuan Tan BX, Tan EY, Luo KQ (2016). High expression of MnSOD promotes survival of circulating breast cancer cells and increases their resistance to doxorubicin. Oncotarget.

[8] Liu Z, Chen J, Ren Y, Liu S, Ba Y, Zuo A, Luo P (2024). Multi-stage mechanisms of tumor metastasis and therapeutic strategies. Sig Transduct Target Ther.

[9] Mathew M, Nguyen N, Bhutia Y, Sivaprakasam S, Ganapathy V (2024). Metabolic signature of warburg effect in cancer: an effective and obligatory interplay between nutrient transporters and catabolic/anabolic pathways to promote tumor growth. Cancers.

[10] Ward PS, Thompson CB (2012). Metabolic reprogramming: a cancer hallmark even Warburg did not anticipate. Cancer Cell.

[11] Pascale RM, Calvisi DF, Simile MM, Feo CF, Feo F (2020). The Warburg effect 97 years after its discovery. Cancers.

[12] Birsoy K, Possemato R, Lorbeer FK, Bayraktar EC, Thiru P, Yucel B, Wang T (2014). Metabolic determinants of cancer cell sensitivity to glucose limitation and biguanides. Nature.

[13] Schoeppe R, Babl N, Decking SM, Schönhammer G, Siegmund A, Bruss C, Dettmer K (2023). Glutamine synthetase expression rescues human dendritic cell survival in a glutamine-deprived environment. Front Oncol.

[14] Arner EN, Rathmell JC (2023). Metabolic programming and immune suppression in the tumor microenvironment. Cancer Cell.

[15] Hanahan D, Weinberg RA (2011). Hallmarks of cancer: the next generation. Cell.

[16] Siemann DW, Horsman MR (2015). Modulation of the tumor vasculature and oxygenation to improve therapy. Pharmacol Ther.

[17] Park J, Wang L, Ho PC (2022). Metabolic guidance and stress in tumors modulate antigen-presenting cells. Oncogenesis.

[18] Reinfeld BI, Madden MZ, Wolf MM, Chytil A, Bader JE, Patterson AR, Sugiura A (2021). Cell-programmed nutrient partitioning in the tumour microenvironment. Nature.

[19] Choi IA, Umemoto A, Mizuno M, Park-Min KH, Bone metabolism—an underappreciated player.
*
npj Metab Health Dis
* 2024, 2.. https://www.nature.com/articles/s44324-024-00010-9.

[20] Kaczara P, Czyzynska-Cichon I, Kus E, Kurpinska A, Olkowicz M, Wojnar-Lason K, Pacia MZ (2024). Liver sinusoidal endothelial cells rely on oxidative phosphorylation but avoid processing long-chain fatty acids in their mitochondria. Cell Mol Biol Lett.

[21] Bélanger M, Allaman I, Magistretti PJ (2011). Brain energy metabolism: focus on astrocyte-neuron metabolic cooperation. Cell Metab.

[22] Li Y, Zhao L, Li XF (2021). Hypoxia and the tumor microenvironment. Technol Cancer Res Treat.

[23] Viallard C, Larrivée B (2017). Tumor angiogenesis and vascular normalization: alternative therapeutic targets. Angiogenesis.

[24] Zhou H, Lei P, Padera TP (2021). Progression of metastasis through lymphatic system. Cells.

[25] Pereira ER, Jones D, Jung K, Padera TP (2015). The lymph node microenvironment and its role in the progression of metastatic cancer. Semin Cell Dev Biol.

[26] Ju S, Chen C, Zhang J, Xu L, Zhang X, Li Z, Chen Y (2022). Detection of circulating tumor cells: opportunities and challenges. Biomark Res.

[27] Huang Q, Hu X, He W, Zhao Y, Hao S, Wu Q, Li S, Zhang S, and Shi M, Fluid shear stress and tumor metastasis.
*
Am J Cancer Res
* 2018, 8: 763–777. https://pubmed.ncbi.nlm.nih.gov/29888101/.

[28] Bray F, Laversanne M, Sung H, Ferlay J, Siegel RL, Soerjomataram I, Jemal A (2024). Global cancer statistics 2022: GLOBOCAN estimates of incidence and mortality worldwide for 36 cancers in 185 countries. CA Cancer J Clin.

[29] Joyce JA, Pollard JW (2009). Microenvironmental regulation of metastasis. Nat Rev Cancer.

[30] Bussard KM, Gay CV, Mastro AM (2008). The bone microenvironment in metastasis; what is special about bone?. Cancer Metastasis Rev.

[31] Spencer JA, Ferraro F, Roussakis E, Klein A, Wu J, Runnels JM, Zaher W (2014). Direct measurement of local oxygen concentration in the bone marrow of live animals. Nature.

[32] Altorki NK, Markowitz GJ, Gao D, Port JL, Saxena A, Stiles B, McGraw T (2019). The lung microenvironment: an important regulator of tumour growth and metastasis. Nat Rev Cancer.

[33] Kiesel VA, Sheeley MP, Coleman MF, Cotul EK, Donkin SS, Hursting SD, Wendt MK (2021). Pyruvate carboxylase and cancer progression. Cancer Metab.

[34] Muriel P, López-Sánchez P, Ramos-Tovar E (2021). Fructose and the liver. Int J Mol Sci.

[35] Fowle-Grider R, Rowles Iii JL, Shen I, Wang Y, Schwaiger-Haber M, Dunham AJ, Jayachandran K (2024). Dietary fructose enhances tumour growth indirectly via interorgan lipid transfer. Nature.

[36] Wen J, Yu JZ, Liu C, Ould Ismail AAO, Ma W (2024). Exploring the molecular tumor microenvironment and translational biomarkers in brain metastases of non-small-cell lung cancer. Int J Mol Sci.

[37] Pliszka M, Szablewski L (2021). Glucose transporters as a target for anticancer therapy. Cancers.

[38] Gyimesi G, Pujol-Giménez J, Kanai Y, Hediger MA (2020). Sodium-coupled glucose transport, the SLC5 family, and therapeutically relevant inhibitors: from molecular discovery to clinical application. Pflugers Arch-Eur J Physiol.

[39] Holman GD (2020). Structure, function and regulation of mammalian glucose transporters of the SLC2 family. Pflugers Arch.

[40] Wei Z, Liu X, Cheng C, Yu W, Yi P (2021). Metabolism of amino acids in cancer. Front Cell Dev Biol.

[41] Bian Y, Li W, Kremer DM, Sajjakulnukit P, Li S, Crespo J, Nwosu ZC (2020). Cancer SLC43A2 alters T cell methionine metabolism and histone methylation. Nature.

[42] Commisso C, Davidson SM, Soydaner-Azeloglu RG, Parker SJ, Kamphorst JJ, Hackett S, Grabocka E (2013). Macropinocytosis of protein is an amino acid supply route in Ras-transformed cells. Nature.

[43] Recouvreux MV, Commisso C (2017). Macropinocytosis: a metabolic adaptation to nutrient stress in cancer. Front Endocrinol.

[44] Qiu Z, Liu W, Zhu Q, Ke K, Zhu Q, Jin W, Yu S (2022). The role and therapeutic potential of macropinocytosis in cancer. Front Pharmacol.

[45] Commisso C (2019). The pervasiveness of macropinocytosis in oncological malignancies. Phil Trans R Soc B.

[46] Ramirez C, Hauser AD, Vucic EA, Bar-Sagi D (2019). Plasma membrane V-ATPase controls oncogenic RAS-induced macropinocytosis. Nature.

[47] Olivares O, Mayers JR, Gouirand V, Torrence ME, Gicquel T, Borge L, Lac S (2017). Collagen-derived proline promotes pancreatic ductal adenocarcinoma cell survival under nutrient limited conditions. Nat Commun.

[48] Pechincha C, Groessl S, Kalis R, de Almeida M, Zanotti A, Wittmann M, Schneider M (2022). Lysosomal enzyme trafficking factor LYSET enables nutritional usage of extracellular proteins. Science.

[49] Jayashankar V, Edinger AL (2020). Macropinocytosis confers resistance to therapies targeting cancer anabolism. Nat Commun.

[50] Keenan MM, Chi JT (2015). Alternative fuels for cancer cells. Cancer J.

[51] de la Cruz-López KG, Castro-Muñoz LJ, Reyes-Hernández DO, García-Carrancá A, Manzo-Merino J (2019). Lactate in the regulation of tumor microenvironment and therapeutic approaches. Front Oncol.

[52] Certo M, Tsai CH, Pucino V, Ho PC, Mauro C (2021). Lactate modulation of immune responses in inflammatory versus tumour microenvironments. Nat Rev Immunol.

[53] Harmon C, Robinson MW, Hand F, Almuaili D, Mentor K, Houlihan DD, Hoti E (2019). Lactate-mediated acidification of tumor microenvironment induces apoptosis of liver-resident NK cells in colorectal liver metastasis. Cancer Immunol Res.

[54] Quinn Iii WJ, Jiao J, TeSlaa T, Stadanlick J, Wang Z, Wang L, Akimova T (2020). Lactate limits T cell proliferation via the NAD(H) redox state. Cell Rep.

[55] Zhang Y, Peng Q, Zheng J, Yang Y, Zhang X, Ma A, Qin Y (2023). The function and mechanism of lactate and lactylation in tumor metabolism and microenvironment. Genes Dis.

[56] Cheng J, Yan J, Liu Y, Shi J, Wang H, Zhou H, Zhou Y (2023). Cancer-cell-derived fumarate suppresses the anti-tumor capacity of CD8
^+^ T cells in the tumor microenvironment. Cell Metab.

[57] Gudgeon N, Munford H, Bishop EL, Hill J, Fulton-Ward T, Bending D, Roberts J (2022). Succinate uptake by T cells suppresses their effector function via inhibition of mitochondrial glucose oxidation. Cell Rep.

[58] Huang D, Wang Y, Thompson JW, Yin T, Alexander PB, Qin D, Mudgal P (2022). Cancer-cell-derived GABA promotes β-catenin-mediated tumour growth and immunosuppression. Nat Cell Biol.

[59] Notarangelo G, Spinelli JB, Perez EM, Baker GJ, Kurmi K, Elia I, Stopka SA (2022). Oncometabolited-2HG alters T cell metabolism to impair CD8
^+^ T cell function. Science.

[60] Jin R, Neufeld L, McGaha TL (2025). Linking macrophage metabolism to function in the tumor microenvironment. Nat Cancer.

[61] Zhang T, Ren Y, Yang P, Wang J, Zhou H (2022). Cancer-associated fibroblasts in pancreatic ductal adenocarcinoma. Cell Death Dis.

[62] Clay R, Li K, Jin L (2025). Metabolic signaling in the tumor microenvironment. Cancers.

[63] Li X, Wang Z, Zheng Y, Guan Y, Yang P, Chen X, Peng C (2018). Nuclear receptor nur77 facilitates melanoma cell survival under metabolic stress by protecting fatty acid oxidation. Mol Cell.

[64] Liu R, Lee JH, Li J, Yu R, Tan L, Xia Y, Zheng Y (2021). Choline kinase alpha 2 acts as a protein kinase to promote lipolysis of lipid droplets. Mol Cell.

[65] Chen J, Zou L, Lu G, Grinchuk O, Fang L, Ong DST, Taneja R (2022). PFKP alleviates glucose starvation-induced metabolic stress in lung cancer cells via AMPK-ACC2 dependent fatty acid oxidation. Cell Discov.

[66] Yang L, Venneti S, Nagrath D (2017). Glutaminolysis: a hallmark of cancer metabolism. Annu Rev Biomed Eng.

[67] Le A, Lane AN, Hamaker M, Bose S, Gouw A, Barbi J, Tsukamoto T (2012). Glucose-independent glutamine metabolism via TCA cycling for proliferation and survival in B cells. Cell Metab.

[68] Ying M, You D, Zhu X, Cai L, Zeng S, Hu X (2021). Lactate and glutamine support NADPH generation in cancer cells under glucose deprived conditions. Redox Biol.

[69] Yi Y, Wang G, Zhang W, Yu S, Fei J, An T, Yi J (2025). Mitochondrial-cytochrome C oxidase II promotes glutaminolysis to sustain tumor cell survival upon glucose deprivation. Nat Commun.

[70] Li M, Wang Y, Wei X, Cai WF, Wu J, Zhu M, Wang Y (2024). AMPK targets PDZD8 to trigger carbon source shift from glucose to glutamine. Cell Res.

[71] Xiang L, Mou J, Shao B, Wei Y, Liang H, Takano N, Semenza GL (2019). Glutaminase 1 expression in colorectal cancer cells is induced by hypoxia and required for tumor growth, invasion, and metastatic colonization. Cell Death Dis.

[72] Yuan L, Sheng X, Willson AK, Roque DR, Stine JE, Guo H, Jones HM (2015). Glutamine promotes ovarian cancer cell proliferation through the mTOR/S6 pathway. Endocr Relat Cancer.

[73] Jiang J, Batra S, Zhang J (2021). Asparagine: a metabolite to be targeted in cancers. Metabolites.

[74] Yoon I, Nam M, Kim HK, Moon HS, Kim S, Jang J, Song JA (2020). Glucose-dependent control of leucine metabolism by leucyl-tRNA synthetase 1. Science.

[75] Park S, Chang CY, Safi R, Liu X, Baldi R, Jasper JS, Anderson GR (2016). ERRα-regulated lactate metabolism contributes to resistance to targeted therapies in breast cancer. Cell Rep.

[76] Chen YJ, Mahieu NG, Huang X, Singh M, Crawford PA, Johnson SL, Gross RW (2016). Lactate metabolism is associated with mammalian mitochondria. Nat Chem Biol.

[77] Skinner OS, Blanco-Fernández J, Goodman RP, Kawakami A, Shen H, Kemény LV, Joesch-Cohen L (2023). Salvage of ribose from uridine or RNA supports glycolysis in nutrient-limited conditions. Nat Metab.

[78] Tabata S, Yamamoto M, Goto H, Hirayama A, Ohishi M, Kuramoto T, Mitsuhashi A (2017). Thymidine catabolism as a metabolic strategy for cancer survival. Cell Rep.

[79] Wang T, Gnanaprakasam JNR, Chen X, Kang S, Xu X, Sun H, Liu L (2020). Inosine is an alternative carbon source for CD8
^+^-T-cell function under glucose restriction. Nat Metab.

[80] Li MX, Wu XT, Jing WQ, Hou WK, Hu S, Yan W (2023). Inosine enhances tumor mitochondrial respiration by inducing Rag GTPases and nascent protein synthesis under nutrient starvation. Cell Death Dis.

[81] Schug ZT, Vande Voorde J, Gottlieb E (2016). The metabolic fate of acetate in cancer. Nat Rev Cancer.

[82] Dhar G, Sen S, Chaudhuri G, Virolle MJ (2015). Acid gradient across plasma membrane can drive phosphate bond synthesis in cancer cells: acidic tumor milieu as a potential energy source. PLoS One.

[83] P.V.F.K.P. Okunieff, Blood Flow, Oxygen and nutrient supply, and metabolic microenvironment of human tumors: a review.
*
Cancer Res
* 1989, 49: 6449–6465. https://pubmed.ncbi.nlm.nih.gov/2684393/.

[84] Lugano R, Ramachandran M, Dimberg A (2020). Tumor angiogenesis: causes, consequences, challenges and opportunities. Cell Mol Life Sci.

[85] Mei X, Chen YS, Chen FR, Xi SY, Chen ZP (2017). Glioblastoma stem cell differentiation into endothelial cells evidenced through live-cell imaging. Neuro Oncol.

[86] Angara K, Borin TF, Arbab AS (2017). Vascular mimicry: a novel neovascularization mechanism driving anti-angiogenic therapy (AAT) resistance in glioblastoma. Transl Oncol.

[87] Shaw P, Dwivedi SKD, Bhattacharya R, Mukherjee P, Rao G (2024). VEGF signaling: role in angiogenesis and beyond. Biochim Biophys Acta Rev Cancer.

[88] Pathak A, Pal AK, Roy S, Nandave M, Jain K, Yuan S (2024). Role of angiogenesis and its biomarkers in development of targeted tumor therapies. Stem Cells Int.

[89] Xu Y, Yuan FE, Chen QX, Liu BH (2018). Molecular mechanisms involved in angiogenesis and potential target of antiangiogenesis in human glioblastomas. Glioma.

[90] Jechlinger M (2006). Autocrine PDGFR signaling promotes mammary cancer metastasis. J Clin Invest.

[91] Spring H, Schüler T, Arnold B, Hämmerling GJ, Ganss R (2005). Chemokines direct endothelial progenitors into tumor neovessels. Proc Natl Acad Sci USA.

[92] Nakamura N, Naruse K, Matsuki T, Hamada Y, Nakashima E, Kamiya H, Matsubara T (2009). Adiponectin promotes migration activities of endothelial progenitor cells via Cdc42/Rac1. FEBS Lett.

[93] Han X, Wang Q, Fang S, Wang J, Liu F, Zhang J, Jin G (2022). P4HA1 regulates CD31 via COL6A1 in the transition of glioblastoma stem-like cells to tumor endothelioid cells. Front Oncol.

[94] Cheng L, Huang Z, Zhou W, Wu Q, Donnola S, Liu JK, Fang X (2013). Glioblastoma stem cells generate vascular pericytes to support vessel function and tumor growth. Cell.

[95] Mao Y, Zhu L, Huang Z, Luo C, Zhou T, Li L, Wang G (2020). Stem-like tumor cells involved in heterogeneous vasculogenesis in breast cancer. Endocr Relat Cancer.

[96] Tang S, Xiang T, Huang S, Zhou J, Wang Z, Xie R, Long H (2016). Ovarian cancer stem-like cells differentiate into endothelial cells and participate in tumor angiogenesis through autocrine CCL5 signaling. Cancer Lett.

[97] Comito G, Calvani M, Giannoni E, Bianchini F, Calorini L, Torre E, Migliore C (2011). HIF-1α stabilization by mitochondrial ROS promotes Met-dependent invasive growth and vasculogenic mimicry in melanoma cells. Free Radical Biol Med.

[98] Lizárraga-Verdugo E, Avendaño-Félix M, Bermúdez M, Ramos-Payán R, Pérez-Plasencia C, Aguilar-Medina M (2020). Cancer stem cells and its role in angiogenesis and vasculogenic mimicry in gastrointestinal cancers. Front Oncol.

[99] Zhu Q, Zhang X, Lu F, Miao S, Zhang C, Liu Z, Gao Z (2024). RUNX1-BMP2 promotes vasculogenic mimicry in laryngeal squamous cell carcinoma via activation of the PI3K-AKT signaling pathway. Cell Commun Signal.

[100] Maraqah HH, Abu-Asab MS, Lee HS, Aboud O (2023). Astrocytoma and glioblastoma IDH1-wildtype cells colonize tumor vessels and deploy vascular mimicry. Ultrastruct Pathol.

[101] Jiang X, Deng X, Wang J, Mo Y, Shi L, Wei F, Zhang S (2022). BPIFB1 inhibits vasculogenic mimicry via downregulation of GLUT1-mediated H3K27 acetylation in nasopharyngeal carcinoma. Oncogene.

[102] Leong SP, Naxerova K, Keller L, Pantel K, Witte M (2022). Molecular mechanisms of cancer metastasis via the lymphatic versus the blood vessels. Clin Exp Metastasis.

[103] Chen Q, Zou J, He Y, Pan Y, Yang G, Zhao H, Huang Y (2022). A narrative review of circulating tumor cells clusters: a key morphology of cancer cells in circulation promote hematogenous metastasis. Front Oncol.

[104] Amintas S, Bedel A, Moreau-Gaudry F, Boutin J, Buscail L, Merlio JP, Vendrely V (2020). Circulating tumor cell clusters: united we stand divided we fall. Int J Mol Sci.

[105] Parlani M, Jorgez C, Friedl P (2023). Plasticity of cancer invasion and energy metabolism. Trends Cell Biol.

[106] Zanotelli MR, Zhang J, Reinhart-King CA (2021). Mechanoresponsive metabolism in cancer cell migration and metastasis. Cell Metab.

[107] Zhang J, Goliwas KF, Wang W, Taufalele PV, Bordeleau F, Reinhart-King CA (2019). Energetic regulation of coordinated leader–follower dynamics during collective invasion of breast cancer cells. Proc Natl Acad Sci USA.

[108] Commander R, Wei C, Sharma A, Mouw JK, Burton LJ, Summerbell E, Mahboubi D (2020). Subpopulation targeting of pyruvate dehydrogenase and GLUT1 decouples metabolic heterogeneity during collective cancer cell invasion. Nat Commun.

[109] Gray KM, Stroka KM (2017). Vascular endothelial cell mechanosensing: new insights gained from biomimetic microfluidic models. Semin Cell Dev Biol.

[110] Ortiz-Otero N, Marshall JR, Lash BW, King MR (2020). Platelet mediated TRAIL delivery for efficiently targeting circulating tumor cells. Nanoscale Adv.

[111] Zhou L, Zhang Z, Tian Y, Li Z, Liu Z, Zhu S (2023). The critical role of platelet in cancer progression and metastasis. Eur J Med Res.

[112] Abdol Razak N, Jones G, Bhandari M, Berndt M, Metharom P (2018). Cancer-associated thrombosis: an overview of mechanisms, risk factors, and treatment. Cancers.

[113] Shao B, Wahrenbrock MG, Yao L, David T, Coughlin SR, Xia L, Varki A (2011). Carcinoma mucins trigger reciprocal activation of platelets and neutrophils in a murine model of Trousseau syndrome. Blood.

[114] Zhang W, Zhou H, Li H, Mou H, Yinwang E, Xue Y, Wang S (2023). Cancer cells reprogram to metastatic state through the acquisition of platelet mitochondria. Cell Rep.

[115] Yu L, Guo Y, Chang Z, Zhang D, Zhang S, Pei H, Pang J (2021). Bidirectional interaction between cancer cells and platelets provides potential strategies for cancer therapies. Front Oncol.

[116] Lee C, Jeong S, Jang C, Bae H, Kim YH, Park I, Kim SK (2019). Tumor metastasis to lymph nodes requires YAP-dependent metabolic adaptation. Science.

[117] Ubellacker JM, Morrison SJ (2019). Metabolic adaptation fuels lymph node metastasis. Cell Metab.

[118] Zhang C, Liao Y, Liu P, Du Q, Liang Y, Ooi S, Qin S (2020). FABP5 promotes lymph node metastasis in cervical cancer by reprogramming fatty acid metabolism. Theranostics.

[119] Delclaux I, Ventre KS, Jones D, Lund AW (2024). The tumor-draining lymph node as a reservoir for systemic immune surveillance. Trends Cancer.

[120] Rahim MK, Okholm TLH, Jones KB, McCarthy EE, Liu CC, Yee JL, Tamaki SJ (2023). Dynamic CD8
^+^ T cell responses to cancer immunotherapy in human regional lymph nodes are disrupted in metastatic lymph nodes. Cell.

[121] Yaddanapudi K, Stamp BF, Subrahmanyam PB, Smolenkov A, Waigel SJ, Gosain R, Egger ME (2022). Single-cell immune mapping of melanoma sentinel lymph nodes reveals an actionable immunotolerant microenvironment. Clin Cancer Res.

[122] Hood JL, San RS, Wickline SA (2011). Exosomes released by melanoma cells prepare sentinel lymph nodes for tumor metastasis. Cancer Res.

[123] Zhu T, Wang Z, Zou T, Xu L, Zhang S, Chen Y, Chen C (2021). SOAT1 promotes gastric cancer lymph node metastasis through lipid synthesis. Front Pharmacol.

[124] Sun R, Yuan L, Jiang Y, Wan Y, Ma X, Yang J, Sun G (2023). ALKBH5 activates FAK signaling through m6A demethylation in
*ITGB1* mRNA and enhances tumor-associated lymphangiogenesis and lymph node metastasis in ovarian cancer. Theranostics.

[125] Du Q, Liu P, Zhang C, Liu T, Wang W, Shang C, Wu J (2022). FASN promotes lymph node metastasis in cervical cancer via cholesterol reprogramming and lymphangiogenesis. Cell Death Dis.

[126] Endo H, Inoue M (2019). Dormancy in cancer. Cancer Sci.

[127] Pranzini E, Raugei G, Taddei ML (2022). Metabolic features of tumor dormancy: possible therapeutic strategies. Cancers.

[128] Hu W, Lee SML, Bazhin AV, Guba M, Werner J, Nieß H (2023). Neutrophil extracellular traps facilitate cancer metastasis: cellular mechanisms and therapeutic strategies. J Cancer Res Clin Oncol.

[129] Summers MA, McDonald MM, Croucher PI (2020). Cancer cell dormancy in metastasis. Cold Spring Harb Perspect Med.

[130] Bu P, Chen KY, Xiang K, Johnson C, Crown SB, Rakhilin N, Ai Y (2018). Aldolase B-mediated fructose metabolism drives metabolic reprogramming of colon cancer liver metastasis. Cell Metab.

[131] Dupuy F, Tabariès S, Andrzejewski S, Dong Z, Blagih J, Annis MG, Omeroglu A (2015). PDK1-dependent metabolic reprogramming dictates metastatic potential in breast cancer. Cell Metab.

[132] Zhao J, Schlößer HA, Wang Z, Qin J, Li J, Popp F, Popp MC (2019). Tumor-derived extracellular vesicles inhibit natural killer cell function in pancreatic cancer. Cancers.

[133] Chen L, Zheng H, Yu X, Liu L, Li H, Zhu H, Zhang Z (2020). Tumor-Secreted GRP78 promotes the establishment of a pre-metastatic niche in the liver microenvironment. Front Immunol.

[134] Chen F, Han Y, Kang Y (2021). Bone marrow niches in the regulation of bone metastasis. Br J Cancer.

[135] Yagiz K, Rittling SR (2009). Both cell-surface and secreted CSF-1 expressed by tumor cells metastatic to bone can contribute to osteoclast activation. Exp Cell Res.

[136] Sethi N, Dai X, Winter CG, Kang Y (2011). Tumor-derived jagged1 promotes osteolytic bone metastasis of breast cancer by engaging notch signaling in bone cells. Cancer Cell.

[137] Anloague A, Delgado-Calle J (2023). Osteocytes: new kids on the block for cancer in bone therapy. Cancers.

[138] Diedrich JD, Rajagurubandara E, Herroon MK, Mahapatra G, Hüttemann M, Podgorski I (2016). Bone marrow adipocytes promote the Warburg phenotype in metastatic prostate tumors via HIF-1α activation. Oncotarget.

[139] Luo G, He Y, Yu X (2018). Bone marrow adipocyte: an intimate partner with tumor cells in bone metastasis. Front Endocrinol.

[140] Kakhki VRD, Anvari K, Sadeghi R, Mahmoudian AS, Torabian-Kakhki M (2013). Pattern and distribution of bone metastases in common malignant tumors. Nucl Med Rev.

[141] Tyagi A, Wu SY, Watabe K (2022). Metabolism in the progression and metastasis of brain tumors. Cancer Lett.

[142] Ferraro GB, Ali A, Luengo A, Kodack DP, Deik A, Abbott KL, Bezwada D (2021). Fatty acid synthesis is required for breast cancer brain metastasis. Nat Cancer.

[143] Neman J, Termini J, Wilczynski S, Vaidehi N, Choy C, Kowolik CM, Li H (2014). Human breast cancer metastases to the brain display GABAergic properties in the neural niche. Proc Natl Acad Sci USA.

[144] Busatto S, Yang Y, Walker SA, Davidovich I, Lin WH, Lewis-Tuffin L, Anastasiadis PZ (2020). Brain metastases-derived extracellular vesicles induce binding and aggregation of low-density lipoprotein. J Nanobiotechnol.

[145] Greelish JP, Friedberg JS (2000). Secondary pulmonary malignancy. Surg Clin North Am.

[146] Liu Y, Gu Y, Han Y, Zhang Q, Jiang Z, Zhang X, Huang B (2016). Tumor exosomal RNAs promote lung pre-metastatic niche formation by activating alveolar epithelial TLR3 to recruit neutrophils. Cancer Cell.

[147] Li P, Lu M, Shi J, Gong Z, Hua L, Li Q, Lim B (2020). Lung mesenchymal cells elicit lipid storage in neutrophils that fuel breast cancer lung metastasis. Nat Immunol.

[148] Elia I, Rossi M, Stegen S, Broekaert D, Doglioni G, van Gorsel M, Boon R (2019). Breast cancer cells rely on environmental pyruvate to shape the metastatic niche. Nature.

[149] Elia I, Broekaert D, Christen S, Boon R, Radaelli E, Orth MF, Verfaillie C (2017). Proline metabolism supports metastasis formation and could be inhibited to selectively target metastasizing cancer cells. Nat Commun.

[150] Wang Y, Li Y, Zhong J, Li M, Zhou Y, Lin Q, Zong S (2023). Tumor-derived Cav-1 promotes pre-metastatic niche formation and lung metastasis in breast cancer. Theranostics.

[151] Mao X, Tey SK, Yeung CLS, Kwong EML, Fung YME, Chung CYS, Mak LY (2020). Nidogen 1-enriched extracellular vesicles facilitate extrahepatic metastasis of liver cancer by activating pulmonary fibroblasts to secrete tumor necrosis factor receptor 1. Adv Sci.

[152] Sivanand S, Gultekin Y, Winter PS, Vermeulen SY, Tchourine KM, Abbott KL, Danai LV (2024). Cancer tissue of origin constrains the growth and metabolism of metastases. Nat Metab.

[153] Wang Z, Wang Y, Li Z, Xue W, Hu S, Kong X (2023). Lipid metabolism as a target for cancer drug resistance: progress and prospects. Front Pharmacol.

[154] Butler M, van der Meer LT, van Leeuwen FN (2021). Amino acid depletion therapies: starving cancer cells to death. Trends Endocrinol Metab.

[155] Shen YA, Chen CL, Huang YH, Evans EE, Cheng CC, Chuang YJ, Zhang C (2021). Inhibition of glutaminolysis in combination with other therapies to improve cancer treatment. Curr Opin Chem Biol.

[156] Cervantes-Madrid D, Dominguez-Gomez G, Gonzalez-Fierro A, Perez-Cardenas E, Taja-Chayeb L, Trejo-Becerril C, Duenas-Gonzalez A (2017). Feasibility and antitumor efficacy in vivo, of simultaneously targeting glycolysis, glutaminolysis and fatty acid synthesis using lonidamine, 6-diazo-5-oxo-L-norleucine and orlistat in colon cancer. Oncol Lett.

[157] Wind S, Schmid U, Freiwald M, Marzin K, Lotz R, Ebner T, Stopfer P (2019). Clinical pharmacokinetics and pharmacodynamics of nintedanib. Clin Pharmacokinet.

[158] Serova M, Tijeras-Raballand A, Santos CD, Martinet M, Neuzillet C, Lopez A, Mitchell DC (2016). Everolimus affects vasculogenic mimicry in renal carcinoma resistant to sunitinib. Oncotarget.

[159] Ito T, Handa H (2020). Molecular mechanisms of thalidomide and its derivatives. Proc Jpn Acad Ser B.

[160] Li Z, Wang Q, Huang X, Yang M, Zhou S, Li Z, Fang Z (2023). Lactate in the tumor microenvironment: a rising star for targeted tumor therapy. Front Nutr.

[161] McBrayer SK, Cheng JC, Singhal S, Krett NL, Rosen ST, Shanmugam M (2012). Multiple myeloma exhibits novel dependence on GLUT4, GLUT8, and GLUT11: implications for glucose transporter-directed therapy. Blood.

[162] Rohaan MW, Borch TH, van den Berg JH, Met Ö, Kessels R, Geukes Foppen MH, Stoltenborg Granhøj J (2022). Tumor-infiltrating lymphocyte therapy or ipilimumab in advanced melanoma. N Engl J Med.

[163] Schwarz S, Gockel LM, Naggi A, Barash U, Gobec M, Bendas G, Schlesinger M (2020). Glycosaminoglycans as tools to decipher the platelet tumor cell interaction: a focus on P-selectin. Molecules.

[164] Restivo A, Cocco IMF, Casula G, Scintu F, Cabras F, Scartozzi M, Zorcolo L (2015). Aspirin as a neoadjuvant agent during preoperative chemoradiation for rectal cancer. Br J Cancer.

[165] Zhan Q, Liu B, Situ X, Luo Y, Fu T, Wang Y, Xie Z (2023). New insights into the correlations between circulating tumor cells and target organ metastasis. Sig Transduct Target Ther.

[166] Wang M, Xia F, Wei Y, Wei X (2020). Molecular mechanisms and clinical management of cancer bone metastasis. Bone Res.

[167] Bose S, Allen AE, Locasale JW (2020). The molecular link from diet to cancer cell metabolism. Mol Cell.

[168] Castejón M, Plaza A, Martinez-Romero J, Fernandez-Marcos PJ, de Cabo R, Diaz-Ruiz A (2020). Energy restriction and colorectal cancer: a call for additional research. Nutrients.

[169] Nencioni A, Caffa I, Cortellino S, Longo VD (2018). Fasting and cancer: molecular mechanisms and clinical application. Nat Rev Cancer.

[170] Roy J, Watson JE, Hong IS, Fan TM, Das A (2018). Antitumorigenic properties of omega-3 endocannabinoid epoxides. J Med Chem.

